# American Gynecological Society

**Published:** 1905-07

**Authors:** 


A Monthly Review of Medicine and Surgery.

*EDITOR:*

WILLIAM WARREN POTTER, M. D.

All communications, whether of a literary or business nature, books for review and exchanges, should be addressed to the editor 284 Franklin St., Buffalo, N. Y.

Vol. XLIV.—Lx. JULY, 1905. No. 12

### American Gynecological Society.

THIS famous society held its thirtieth annual meeting at Niagara Falls, May 25-27, 1905, during which it disposed of an unusually instructive and interesting program. The social features of the occasion, besides the usual trolley excursions around the cataract environment to Queenston and the Gorge tour, included a reception on Thursday evening, May 25, by Dr. and Mrs. E. C. Dudley, and a smoker on Friday evening, given by Drs. Mann, Frederick, Ford, Van de Warker, and Robb,—all of which were numerously attended and greatly enjoyed.

The reception by the president and his charming wife, after the preliminaries were met, partook of the nature of a musicale, with Mrs. Evelyn Choate at the piano, Mr. Charles McCreary, baritone, and Dr. J. O. Frankenstein, tenor. Classic solos and duets, vocal and instrumental, were rendered and the artists received the generous encores of their charmed listeners.

### THE PRESIDENT'S ADDRESS.

One of the special features of the scientific part of the meeting was the annual address of the president, Dr. E. C. Dudley, of Chicago. For many years Dr. Dudley has occupied a place in the front rank of the profession as a gynecologist, teacher, and author, his textbook on gynecelogy being recognised as the best practical or working treatise yet published in America. Therefore, it was fitting that he should speak for the gynecological profession in clear tones and plain language in refutation of some of the aspersions that have been cast against it both by gynecologists and others. Particularly did he emphasise the fact that the "passing of gynecology" has not taken place, and he warned all prospectors, with dog, pick, and gun to "keep off" the claim.

But as we give below what he said in his own language, which is a forceful presentation of the case and deserves the careful reading of all gynecologists, we need not further amplify.

THE EXPANSION OF GYNECOLOGY.

The general law that progress in any direction is characterised by specialisation, with its attendant classification and simplicity, has been exemplified in no great movement more strongly than in the development of the medical profession during the last three decades. The late Samuel D. Gross, foremost general surgeon of his day, after a long period of active service as author, teacher, and practitioner, writing the preface to the sixth edition of his System of Surgery in October, 1882, thus early gives credit to specialisation for the unparallel advances in modern surgery. Specialisation, he says in substance, has penetrated with its methods and instruments of research the innermost recesses of the human body, and in a comparatively brief period has achieved triumphs which general surgery perhaps never would have accomplished.

In the earlier period when the specialist confined himself to a particular organ, disregarding its relations to the general system, when frequently exclusive books appeared from this author, for example, on the stomach, or from that one on the brain, specialisation was cumbersome, narrow, ineffective and a hindrance to scientific medicine. Finally, the logical tendency to study each part not by itself but in its essential relations to the whole system, gave rise to such a welding together into a great unit of all the specialties, that any organ even though recognised in its individual importance and autonomy at the same time was equally recognised as subject to general law. It then became apparent that physiological and pathological processes, such as circulation and infection, were substantially the same whatever the organ involved. Order then came out of chaos and specialisation became a potent factor in the simplification and progress of medicine. From this time forward laryngology, rhinology, orthopedics, ophthalmology, neurology, chimatology, state medicine, obstetrics and gynecology rapidly developed and became identified in all parts of the civilised world with remarkable groups of men who have strengthened scientific medicine by building up these departments to an extent unequaled in any other period of history.

In nearly all of the medical teaching centers of America and Europe the most conspicuous specialty of modern medicine, gynecology, has enjoyed full recognition not only in the day of its early struggle, but later in the period of its highest development, with the significant result that in dignity of position and in output of scientific product it has in many respects outbalanced the department of general surgery itself.

The progress of gynecology has been marked by two pronounced periods: The first was an earlier period, characterised by great activity in the perfection of numerous plastic operations on the vaginal side of the pelvic floor. This development of minor plastic surgery calls to mind many familiar names at home and abroad, most conspicuous among them the names of two of our founders, Emmet and Marion-Sims. The second or later period was one of tremendous progress in the surgery of the obverse abdominal side of the pelvic floor. Now a third period is before us in which gynecology has taken to itself the whole field of abdominal surgery.

The early gynecologist was logically led by the anatomical, physiological, and pathological unity of the reproductive organs into the peritoneal surgery of the pelvic cavity and thence by anatomic continuity into that of the upper abdomen. In peritoneal surgery, his educated touch, his special surgical judgment, and, above all, his training in the technic of plastic gynecology, placed him on a decided vantage ground over the general surgeon.

This widening of limitations to include the territory of abdominal surgery has given rise to an extraordinary and altogether interesting effort on the part of the general surgeon to promulgate the erroneous idea that the gynecologist has become a general surgeon and thereby has forced gynecology as a specialty into the background, where any one, even without special preparation, may practise it. Thus we hear of "The Merging of Gynecology into General Surgery." "The Passing of a Great Specialty.'" "The Expansion or Obliteration of a Specialty." We are told that gynecology is a finished subject, and that "he who runs may read," that it is only a matter of a few operative procedures, that the technic of it is now perfected and ready-made for the hand of any one, that soon in medical journals, in textbooks, in medical schools, in societies and hospitals, gynecology will be merged into general surgery and the name will be forgotten.

In considering this most recent attitude toward gynecology I do not refer to the practitioner who may be so situated that the most competent experts are not available; necessarily he may be compelled to the best of his ability, not for himself alone, but in the interest of his patient, to undertake not only gynecology, but all the other specialties, nor do I deny that a general surgeon of sufficient versatility may carry on miscellaneous surgical work and at the same time if he will undergo the necessary long and careful training, may acquire the special judgment, the special diagnostic and operative technic essential to proficiency in the practice of a great specialty; but this admission does not weaken the indictment which I would offer against a type of general surgeon, whose number increases day by day, whose relation to this specialty is the outcome of a reasoning all his own, a reasoning from the plausible premise that "the gynecologist, having perfected and simplified his specialty, has found it too narrow and has expanded" to the specious conclusion that gynecology is an insignificant branch and that the gynecologist therefore has undertaken general surgery. This logic gives rise to a sophistry; if the gynecologist is a general surgeon, conversely the general surgeon is a gynecologist. As the times change and we change with them, this type of universal operator, quick to seize on and to turn to his own account the intimation that this specialty has passed, with refinement neither of diagnostic nor operative technic, with no appreciation of his limitations, hypnotised by an apprenticeship of six weeks in some postgraduate school, or by no apprenticeship at all, emboldened by the fact that no one has called him to account, would make gynecology crude and common, would persuade the public and the profession that it is a mere caudal appendix to surgery on which no one fears to tread. Let us for the moment dismiss the general discussion of the subject and imagine a private hospital conducted under certain practical conditions of business management and promotion, with a year of active practice in capital operations, most of them belonging to this, forsooth, insignificant branch of surgery and a mortality of 70 per cent., and then with this experience as a background, going on for an additional few weeks to eleven more consecutive abdominal operations and 100 per cent. of mortality. Does this sound like a made up story? On the contrary, it is an extreme but nevertheless historical example taken not from the dark ages of surgery but from our own times.

Gynecology has not passed. We are not general surgeons. We are specialists in the diseases of women, and as our later transactions abundantly show, we are to a rapidly increasing extent specialists also in the wider field of abdominal surgery, a field in which the account on the ledger as it stands today will show general surgery indebted to us for a great part of its practical and scientific progress; the claim is valid, for we were blazing the trail through this territory when it was an untrodden wilderness, and it is ours by right of discovery; we were giving laws to govern the conduct of the stranger in this field when it was unknown and unconquered, and it is ours by right of conquest; we received from the pioneers, our teachers, some of whom are with us now, the principles and precepts on which has been built up this most aggressive department of surgery, and it is ours by right of inheritance.

Marion-Sims was not a general surgeon when he laid down the laws which today govern the surgery of the gallbladder, when he foreshadowed the modern treatment of gun shot wounds of the abdomen and thereby set in motion a tide of general abdominal surgery of which the ebb flow, particularly in the upper zones of the abdomen, where we have joined hands with the general surgeon, is already overdue. Do the traditions which properly belong to us count for nothing? Shall we retire in the background? Shall we organise a society of the Cincinnati, enter into our second childhood and live on the memories of the past? Is our work done? Shall we say, "Troy has been, we have been Trojans?" If our work is done, why should we not go at once into voluntary liquidation? Why hold another meeting? But so long as in the diseases of women there are more practical and scientific problems to be solved than we have time to enumerate, our work is not done. Does not the increased strain of modern life notwithstanding improved knowledge of sanitation and hygiene bring about exaggerations of pathology which will demand not less but more of the gynecologist? If we do not respect our own specialty, who will? Let us consider, for example, the every-day subject of dysmenorrhea, about which as yet we know but little; the causes of eclampsia, of which we know less; the purpose of menstruation of which we know nothing; the unknown conditions, which in one case will supply defense against general septic peritonitis and in another apparently similar case will open the way to a rapidly fatal peritoneal infection. Let us reflect that we have not spoken the last word on the surgical treatment of descent, retroversion and other deviations of the pelvic organs; let us consider whether in the next thirty years we or the general surgeons are going to make such improvements in practical gynecology that the hysteropexies, the hysterorrhaphies, the suspensions, the fixations and a number of other procedures may look to our successors as crude and irrational as the clamp and routine use of the drainage tube in ovariotomy look to us at the present time.

The distinguished president then took up the subject of incontinence of urine in women and detailed a surgical procedure for its relief. The following officers were elected: president, Richard B. Maury, Memphis; first vice-president, Howard A. Kelly, Baltimore; second vice-president, Reuben Peterson, Ann Arbor; secretary, J. Riddle Goffe, New York; members of the council, J. Montgomery Baldy, Philadelphia, and E. C. Dudley, Chicago. The next annual meeting will be held at Hot Springs, Va.

